# Arginine Transporters in Human Cancers: Emerging Mechanisms and Clinical Implications

**DOI:** 10.3390/biom16010132

**Published:** 2026-01-12

**Authors:** Xi Cai, Li Shang, Yueshuo Li, Ya Cao, Feng Shi

**Affiliations:** 1 Key Laboratory of Carcinogenesis and Cancer Invasion of Chinese Ministry of Education, Xiangya Hospital, Central South University, Changsha 410078, China; 2 Department of Pathology, National Clinical Research Center for Geriatric Disorders, Xiangya Hospital, Central South University, Changsha 410078, China; 3 Key Laboratory of Carcinogenesis of National Health Commission, Cancer Research Institute, Xiangya School of Basic Medical Sciences, Central South University, Changsha 410078, China

**Keywords:** arginine, arginine transporter, cancer, metabolism

## Abstract

Arginine is a semi-essential amino acid for adults, which serves as a central hub synthesizing various metabolites. Arginine plays a critical role in carcinogensis. As a polar amino acid, the uptake and the transportation of arginine across cell membrane systems rely on transporter proteins. Arginine transporters remain critically important, particularly as potential biomarkers and therapeutic targets in cancer. Based on the subcellular localization, arginine transporters could be divided into two types: cell membrane arginine transporters and intracellular membrane arginine transporters. This review aims to investigate the latest advancements of arginine transporter proteins in cancer, focusing on their cellular localization, structural characteristics, and mechanism, with the goal of promoting the design and development of targeted anticancer therapeutics against these transporters.

## 1. Introduction

Under physiological conditions, arginine from endogenous synthesis is usually sufficient to meet the needs of adults. In infancy, the amount of endogenously synthesized arginine cannot satisfy the requirement, and additional dietary supplements are needed. Therefore, arginine is a semi-essential amino acid for adults [1,2,3].

**Figure 1:**
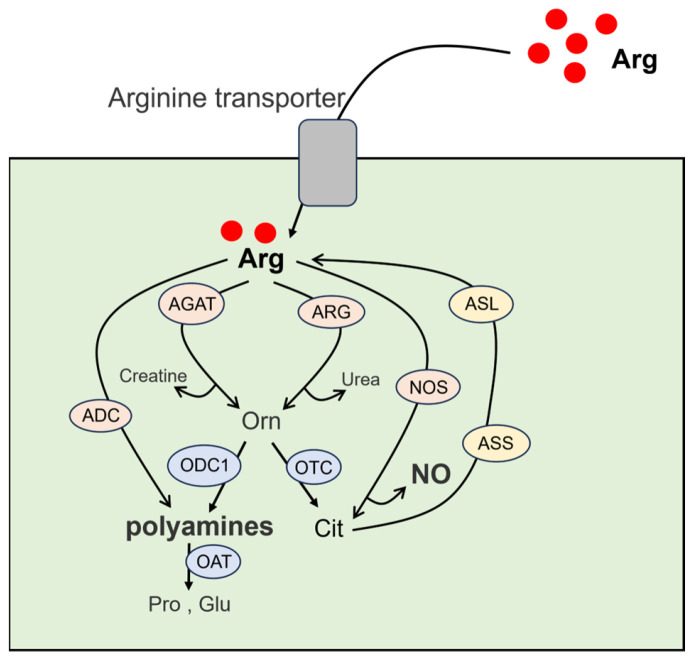
Schematic diagram of arginine metabolism. Following cellular uptake via the arginine transporter, arginine undergoes metabolism through several pathways. It can be catalyzed by nitric oxide synthase (NOS) into NO and citrulline, or by ARG into ornithine and urea, or by L-arginine:glycine amidinotransferase (AGAT) into ornithine and homoarginine or creatine. Ornithine generated in these steps has three fates: decarboxylation by ornithine decarboxylase 1 (ODC1) to form polyamines including putrescine, spermidine, and spermine; transamination by ornithine aminotransferase (OAT) to produce proline or glutamate; or conversion to citrulline by ornithine transcarbamylase (OTC). Citrulline, a product of these reactions, is recycled back to arginine via ASS1 and ASL, which is a cycle in most mammalian cells. In addition to polyamine synthesis from ornithine, arginine can also be converted to polyamines via arginine decarboxylase (ADC) and agmatinase. Arg, arginine; Cit, citrulline; Glu, glutamate; Orn, ornithine; Pro, proline.

There are three main sources of arginine, including dietary intake, protein breakdown, and endogenous synthesis [1,2]. The biosynthesis of arginine is primarily catalyzed by argininosuccinate synthetase 1 (ASS1, the central rate-limiting enzyme) and argininosuccinate lyase (ASL), via the intestine–kidney axis (Figure 1). In kidney cells, citrulline absorbed from the small intestine is employed as the substrate of arginine biosynthesis [1,4,5]. Arginine serves as a central hub for the synthesis of various metabolites (Figure 1), which can be utilized for the synthesis of proteins, nitric oxide (NO), citrulline, ornithine, urea, homoarginine, creatine, polyamines, proline, glutamate, and agmatine, among others [2,6]. Research indicates that arginine and its metabolic intermediates can promote the growth of certain tumors [7,8]. Especially, arginine is able to hyperactivate mTORC1 signaling, which thereby promotes cellular growth and metabolism [9].

**Figure 2:**
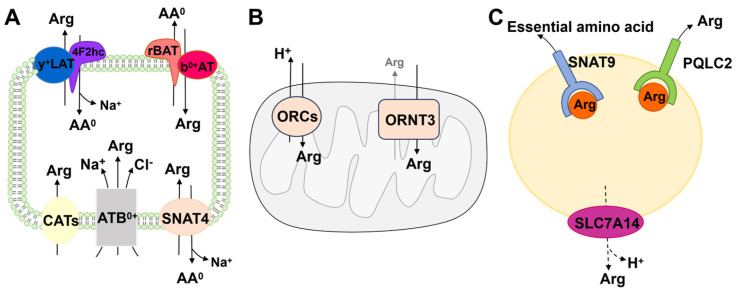
Physiology of arginine transporters. Schematic representation of transport systems for arginine and Co-transport substrate in hypothetical cell membrane and endocytosis ((**A**), cell; (**B**), mitochondria; (**C**), lysosome). y+LAT1/2 and 4F2hc, cationic and neutral amino acids; b0,+AT and rBAT, cationic and neutral amino acids; CATs, cationic amino acid transporters; ATB0,+, a transmembrane glycoprotein; SNAT4, neutral amino acid transporter 4; ORCs and ORNT3, mitochondrial cationic amino acid transporter; SNAT9 and PQCL2, lysosomal membrane transporter; AA0, neutral amino acids.

Arginine can be exchanged between different tissues and cellular compartments. As a polar amino acid, the uptake and the transportation of arginine across cell membrane systems rely on transporter proteins [10,11]. These transporter proteins in mammalian cells belong to the Solute Carrier (SLC) gene family at the molecular level, including SLC3, SLC6, SLC7, SLC15, SLC25, SLC38, and SLC66 families, while SLC15 remains to be confirmed. Members of the SLC7 family are generally considered to be the primary arginine transporters [12,13]. In addition, based on the subcellular localization, arginine transporters could be divided into two types: cell membrane arginine transporters and intracellular membrane arginine transporters (Figure 2, Table 1 and Table 2).

This review aims to investigate the latest advancements of arginine transporter proteins in cancer, focusing on their cellular localization, structural characteristics, and mechanism, with the goal of promoting the design and development of targeted anticancer therapeutics against these transporters.

## 2. Cell Membrane Arginine Transporters

### 2.1. Cationic Amino Acid Transporter (CAT)

**Figure 3:**
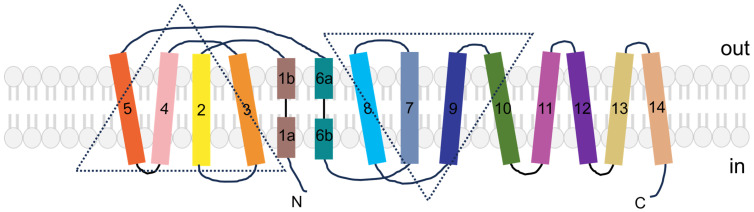
Topological diagram of the SLC7 and SLC6 subfamilies. These transporters consist of two homologous core domains, each containing five transmembrane regions (TMs), namely TMs 1–5 and TMs 6–10, which are arranged with a pseudo-two-fold axis of symmetry and marked with two dashed triangular symbols. The initial TM in the two inverted repeats (TM1 and TM6) are discontinuous, composed of two short α-helices designated as TM1a, TM1b and TM6a, TM6b. TMs 11 and 12, which are present in L-type amino acid transporters (LATs) and cationic amino acid transporters (CATs), diverge from the inverted repeat pattern and form a V-shaped configuration on the extracellular side of TM10. In addition, the first 12 transmembrane regions share high structural similarity. TMs 13/14 are uniquely present in CATs but absent in any other members of the family. Therefore, CATs are categorized as 14-transmembrane-domain proteins containing TMs 13/14, whereas LATs and heteromeric amino acid transporters (HATs) are 12-transmembrane-domain proteins that only possess the first 12 TMs.

**Figure 4:**
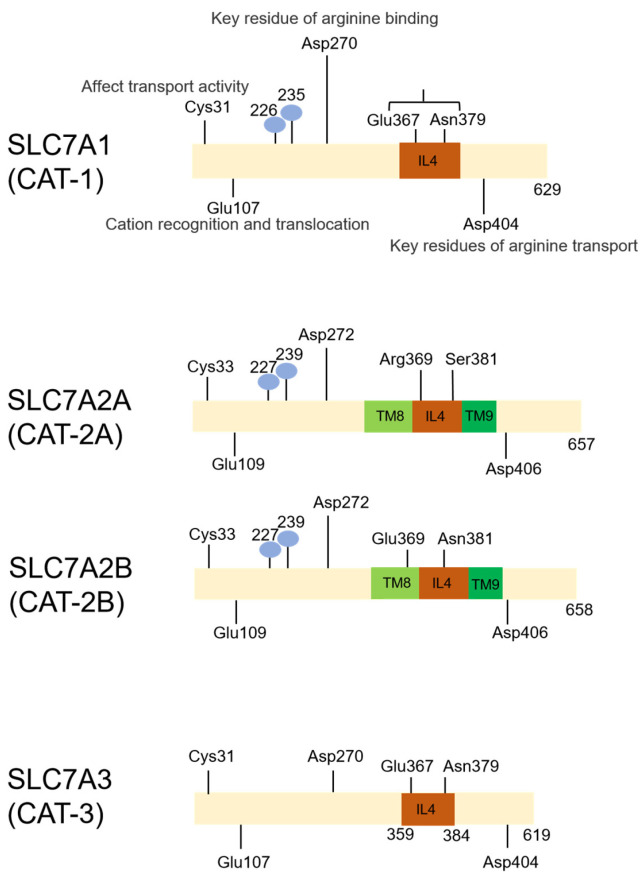
The primary structure diagram of SLC7A1–3 (CAT-1/2/3). All the aforementioned loci are conserved within the CAT enzyme family, exhibiting similar or analogous functions. Notably, two amino acid sites Arg369 and Ser381 in CAT-2A are critical determinants of its low substrate affinity, whereas the Glu-Asn pairs in CAT-1, -2B, and -3 primarily confer high substrate affinity. TM, transmembrane region; IL, intracellular ring; blue spheres, N-glycosylation sites; red dotted box, the difference between CAT-2A and CAT-2B is only 42 amino acids.

Arginine is a cationic amino acid. Cationic amino acid transporters (CATs) belong to the SLC7 subfamily, including SLC7A1 (CAT-1), SLC7A2 (CAT-2A/B), SLC7A3 (CAT-3), SLC7A4 (CAT-4), and SLC7A14. They are characterized by 14 predicted transmembrane domains with both N- and C-termini oriented intracellularly (Figure 3). There is a high sequence homology among SLC7A1–3 (Figure 4), which are localized on the cell membrane [14,15]. These CATs are the primary transporters for the uptake of cationic amino acids from the extracellular space via a sodium-independent transport system [15,16]. SLC7A4 is expressed in the cytoplasm, which lacks amino acid transport activity [17]. SLC7A14 is located on the membrane of lysosomes, which will be discussed in the section of “intracellular membrane transport proteins”.

#### 2.1.1. SLC7A1 (CAT-1)

SLC7A1 is constitutively expressed in human tissues, which plays a critical role in the uptake of ornithine, arginine, and lysine (Figure 3 and Figure 4) [18]. SLC7A1 may favor angiogenesis by enhancing the synthesis of NO in an arginine-dependent way [19]. Overexpression of SLC7A1 could regulate the tumor microenvironment [20,21].

CAT-1 protein expression is elevated in colorectal cancer cells and extracellular vesicles derived from these cells. Extracellular-vesicle-mediated transfer of CAT-1 enhances vascular endothelial cell growth and tubule formation by promoting arginine transport [21]. CAT-1 is also highly expressed in ovarian cancer tissues, which is associated with poorer survival outcomes. Overexpression of SLC7A1 promotes tumor development and cisplatin resistance by regulating the metabolic remodeling of free amino acids [22].

#### 2.1.2. SLC7A2 (CAT-2A/B)

There are two splice variants of SLC7A2, namely CAT-2A and CAT-2B (Figure 3 and Figure 4) [15]. CAT-2A is constitutively expressed in human cells, while CAT-2B expression may be typically induced by inflammatory cytokines or polysaccharides. CAT-2A exhibits a low affinity to cationic amino acids, while the affinity of CAT-2B is relatively higher. Moreover, CAT-2B is a known high-affinity transporter of arginine in macrophages [16,23].

In pancreatic ductal adenocarcinoma (PDAC) tissues, the expression of SLC7A2 is upregulated by RIOK3, which enhances the uptake of arginine [24]. Arginine may bind to CASTOR1 and thereby activate mTORC1 [25,26]. The RIOK3/SLC7A2/mTORC1 axis promotes PDAC cell proliferation, invasion, and migration, which provides a potential strategy by targeting arginine metabolism [24].

However, lower SLC7A2 expression is associated with worse prognosis of ovarian cancer, hepatocellular carcinoma (HCC), and non-small cell lung carcinoma. SLC7A2 silencing enhances cell proliferation and drug resistance in non-small cell lung carcinoma [27]. In hepatocellular carcinoma, loss of SLC7A2 impairs arginine transport and the activation of cGMP-dependent protein kinase II (PKG II), which leads to the activation of NF-κB through PI3K/Akt signaling [28,29]. Elevated expression of CXCL1 induced by NF-κB facilitates the recruitment of myeloid-derived suppressor cells (MDSCs) and M2 tumor-associated macrophages (TAMs) and Tregs, which promotes HCC immune escape [29,30,31]. These data suggest that SLC7A2 may act as a tumor suppressor.

#### 2.1.3. SLC7A3 (CAT-3)

CAT-3, encoded by the SLC7A3 gene, is expressed in various regions of the human brain (Figure 3 and Figure 4). And it is extensively expressed in peripheral tissues—highly in the thymus, with stronger expression in the uterus, testes, and mammary glands and weaker expression in the ovaries and stomach. SLC7A3 functions as a unidirectional amino acid transporter [14].

SLC7A3 expression is downregulated in breast cancer tissues, and patients with low SLC7A3 expression reveal a good prognosis in the early stage of breast cancer [32]. However, SLC7A3 may also exert an oncogenic effect in other cancers. In osteosarcoma (OS) and thyroid carcinoma, high expression of SLC7A3 is associated with poor prognosis of patients [33,34]. SLC7A3 enhances arginine uptake and increases intracellular arginine levels. Arginine serves as an effector for mTORC1 activation to promote cell growth in response to glutamine starvation [35]. Arginine is also able to enhance SP1 stability via an ERK-independent pathway, while SP1 promotes SLC7A3 transcription and thereby facilitates arginine uptake in OS cells [33,36]. Intracellular arginine levels also play a role in macrophage reprogramming. By control iNOS-derived NO, arginine facilitates an antitumor M1-like and protumor M2-like phenotype shift, which promotes OS progression [33,37]. SLC7A3-mediated arginine uptake might be used as a target for OS treatment.

### 2.2. Heterodimer Amino Acid Transporter (HAT)

**Figure 5:**
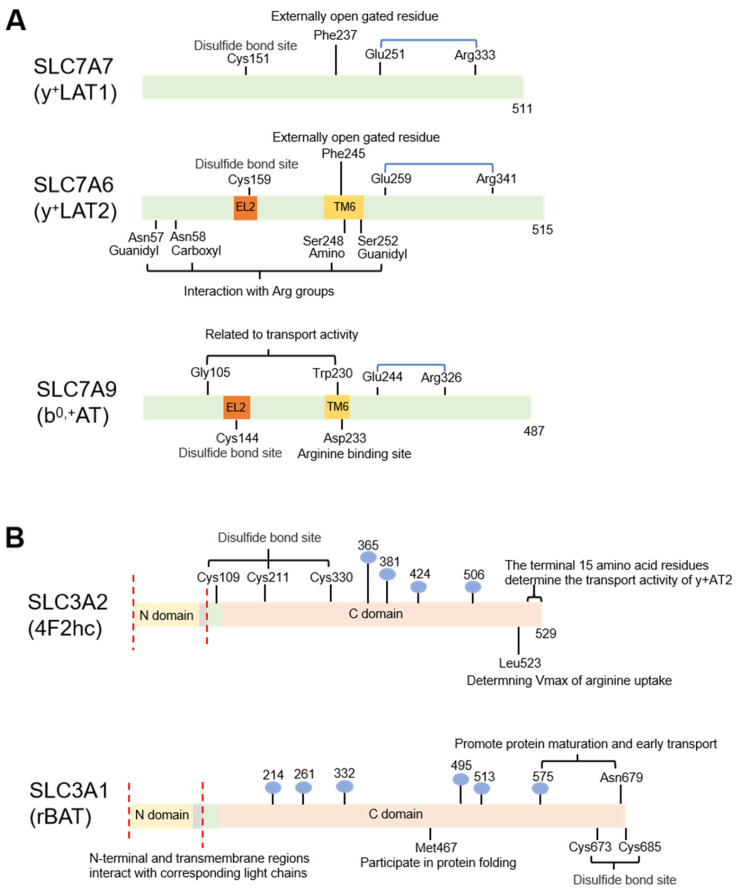
The primary structure diagram of HATs. (**A**) depicts the HAT light subunit, while (**B**) illustrates its heavy subunit. The HAT complex exists as a heterodimer, stabilized by disulfide bonds between the light and heavy subunits. The heavy subunit is a single-pass transmembrane domain primarily responsible for anchoring to the plasma membrane, whereas the light subunit confers transport activity and substrate specificity. In (**A**), the blue brackets indicate salt bridges. In (**B**), the gray region represents the transmembrane domain of the heavy subunit, the green area denotes its neck region, and the blue spheres indicate N-glycosylation sites. TM, transmembrane region; EL, extracellular ring.

The heteromeric amino acid transporter (HAT) comprises two subunits, a light chain and a heavy chain, which are linked by a conserved disulfide bridge [38,39]. The SLC7 family is divided into two subfamilies, CATs and the L-type amino acid transporters (LATs). The light chains of HATs are members of the LAT subfamily, including SLC7A5 (LAT1), SLC7A6 (y+LAT2), SLC7A7 (y+LAT1), SLC7A8 (LAT2), SLC7A9 (b0,+AT), SLC7A10 (alanine–serine–cysteine transporter 1, Asc-1), and SLC7A11 (cystine/glutamate antiporter, xCT) (Figure 3 and Figure 5). The heavy chains belong to the SLC3 family, SLC3A1 (rBAT), and SLC3A2 (4F2hc), which are type II membrane glycoproteins [40,41]. The heavy chain combines with the light one to form a disulfide-linked heterodimer. Usually, the heavy chain is essential for the stability of the light chain and for its localization to the plasma membrane. The light chain is responsible for the transport activity and substrate specificity [41,42]. HATs could transport a broad spectrum of amino acids by multiple combination patterns of these chains, including system L (neutral amino acids, LAT1/2 and 4F2hc), system asc (neutral amino acids, Asc-1 and 4F2hc), system y+L (cationic and neutral amino acids, y+LAT1/2 and 4F2hc), system b0,+ (cationic and neutral amino acids, b0,+AT and rBAT), and system xc− (negatively charged amino acids, xCT and 4F2hc) [43]. Systems y+L and b0,+ play critical roles in arginine transport.

#### 2.2.1. System y+L

y+LAT1 (SLC7A7)/4F2hc (SLC3A2) and y+LAT2 (SLC7A6)/4F2hc (SLC3A2) are the molecular correlates of system y+L (Figure 2 and Figure 5). System y+L is responsible for the influx of neutral amino acids (Na+-dependent) and the efflux of cationic amino acids (Na+-independent). Especially, y+LAT2/4F2hc mediates the efflux of arginine in exchange with glutamine dependent on the presence of Na+ [18,44,45].

- SLC7A7

Activation of transcription factor 3 (ATF3), the transcription factor of SLC7A7, is downregulated in hepatocellular carcinoma, which leads to the transcriptional inhibition of SLC7A7 [46]. Low expression of SLC7A7 decreases arginine efflux and increases intracellular arginine accumulation, which further enhances mTORC1 activation and subsequently promotes lipogenesis and tumor progression [47].

However, SLC7A7 is highly expressed in colorectal cancer and is predictive of a worse prognosis. Mechanistically, SLC7A7 may promote colorectal cancer metastasis through the SLC7A7/APC/Wnt/β-catenin signaling pathway [48].

- SLC7A6

The mRNA expression of Signal Transducer and Activator of Transcription 5A (STAT5A) is relatively downregulated in the majority of human hepatocarcinogenesis tissues, whereas SLC7A6 exhibits higher expression levels in HCC [49]. Various growth factors or hormones activate STAT5A directly via Janus kinases (JAKs) or through alternative pathways [50]. Activated STAT5A suppresses SLC7A6 expression, which leads to the decreased uptake of glutamine, arginine, and other cationic amino acids. The low level of arginine inhibits mTORC1 signaling and subsequent cellular proliferation and metastasis [49].

c-Myc directly binds to the promoter region of SLC7A6, inducing its transcriptional upregulation, which results in a significant increase in glutamine, arginine, and other cationic amino acids’ uptake and activation of the mTORC1 signaling pathway [51]. mTORC1 phosphorylates the eukaryotic translation initiation factor 4E (eIF4E) binding protein 1 (4EBP1), causing its dissociation from eIF4E, thereby regulating the translation of c-Myc, promoting cell cycle progression [52]. Additionally, studies have demonstrated that c-Myc can directly enhance the transcription of eIF4E [53]. These findings suggest the existence of a positive feedback loop involving c-Myc, amplifying the effects of arginine on cellular proliferation and tumor growth [51]. CirSLC7A6 downregulates phosphorylation of AKT, mTOR, ERK, and p38 in ovarian cancer cells, while suppressing Bcl-2 and matrix metalloproteinase 2 (MMP-2) expression. It is hypothesized that CirSLC7A6 significantly downregulates miR-2682-5p and upregulates SLC7A6, thereby enhancing glutamine and arginine transport, activating mTORC1 signaling, and indirectly stimulating the PI3K/AKT and ERK/p38 MAPK pathways. This may increase MMP-2’s expression, promoting cellular proliferation and migration and contributing to chemoresistance [54]. Huang Censu inhibits the CirSLC7A6/miR-2682-5p/SLC7A6 pathway and suppresses amino acid transport compensation, thereby increasing ovarian cancer cell sensitivity to cisplatin and inducing apoptosis [54].

- SLC3A2

SLC3A2 is markedly upregulated in non-small cell lung carcinoma tissues [55], and its knockdown inhibits osteosarcoma proliferation [56]. As an accessory protein of integrins, SLC3A2 mediates integrin-induced phosphorylation of focal adhesion kinase (FAK), thereby activating the PI3K/AKT/mTOR signaling pathway [55,57]. It is also hypothesized that SLC3A2 activates CD98hc through its ligand galectin-3, stimulating the FAK/PI3K/AKT/GSK-3β/β-catenin axis [55], which regulates cell cycle progression and promotes epithelial–mesenchymal transition (EMT).

SLC3A2 is overexpressed in human lung squamous cell carcinoma and lung adenocarcinoma, where it activates the FAK/Ras/Raf/MEK/ERK signaling pathway to induce tumorigenesis, resulting in poor patient prognosis. SLC3A2 mRNA levels are significantly elevated in stage I–IV tumor tissues with no notable difference between the two, indicating that SLC3A2 functions as an initiator of tumorigenesis rather than a promoter of tumor progression [58].

#### 2.2.2. System b0,+

b0,+AT (SLC7A9)/rBAT (SLC3A1) is the molecular correlate of system b0,+, which is responsible for the high-affinity influx of cystine and cationic amino acids and low-affinity efflux of neutral amino acids in a Na+-independent manner (Figure 2 and Figure 5) [45,59]. The system b0,+ is specifically expressed at the apical membrane domains of small intestinal epithelial cells and proximal renal tubules. In these tissues, the apical system b0,+ controls the absorption of cationic amino acids, including arginine, while the basolateral system y+L facilitates arginine efflux [44].

SLC3A1 is overexpressed in breast cancers, which is correlated with advanced clinical stage. Patients with high expression of SLC3A1 may indicate a poor prognosis [60]. Upregulation of SLC3A1 expression increases the stability of β-catenin via PP2AC/AKT/GSK3β axis and thereby promotes tumorigenesis of breast cancer cells [60,61]. SLC3A1 may serve as a potential target for breast cancer therapy.

### 2.3. SLC6A14

**Figure 6:**
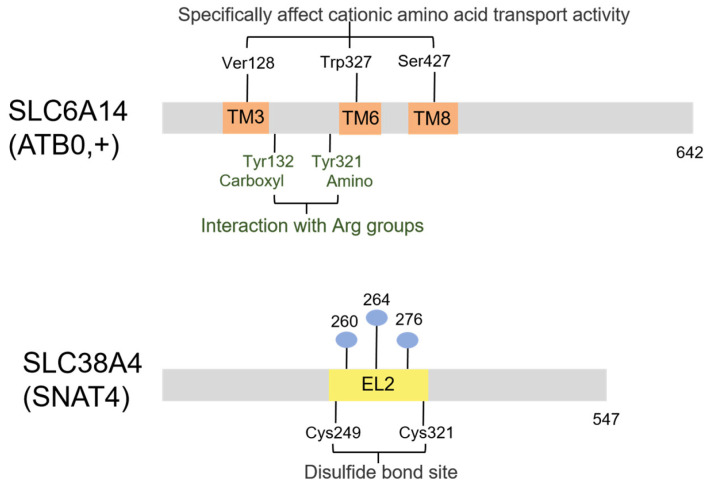
The primary structure diagram of SLC6A14 and SLC38A4. The specificity for arginine of SLC6A14 relies on a binding pocket of key residues, including Ver128 in TM3, Trp327 in TM6, and Ser427 in TM8. Researchers have predicted the binding of Tyr132 and Tyr321 sites to the carboxyl and amino group of arginine, controlling substrate ingress into above binding pocket (green). Meanwhile, there is limited structural information on SLC38A4. Disulfide bonds on SLC38A4 are involved in the transport of L-arginine, and disruption of it impairs transport. The blue spheres indicate N-glycosylation sites. TM, transmembrane region. EL, extracellular ring.

SLC6A14 (ATB0,+) is a transmembrane glycoprotein, which features 12 transmembrane α-helices with both N- and C-termini oriented intracellularly (Figure 3 and Figure 6). This transporter is expressed at the apical membranes of respiratory epithelium, salivary glands, mammary glands, gastric mucosa, pituitary, ocular tissues, and colonic epithelium [62,63]. SLC6A14 controls the Na+/Cl−-coupled concentrative uptake of all neutral amino acids and cationic amino acids, such as arginine and lysine. SLC6A14 catalyzes the extrusion of intracellular Na+/Cl− in exchange for extracellular amino acids, and the exchange ratio is 2:1:1 for Na+/Cl− amino acids. Located on the apical surface, SLC6A14 has a broad substrate specificity and is expressed at significantly higher levels in tumor cells compared to normal cells. So, it is highly suitable for targeted drug delivery [11,64]. Similar to PEPT1, which is situated on the intestinal epithelium, SLC6A14 is extensively utilized in oral drug absorption.

SLC6A14 mRNA expression is significantly upregulated across all four stages (I–IV) of colorectal carcinoma and various histological subtypes [65]. Approximately 85% of sporadic and hereditary colorectal tumors exhibit loss of APC function [66]. The loss of APC may lead to SLC6A14 overexpression, enhancing transport of amino acids such as arginine and activating the mTORC1 pathway, thereby facilitating tumor progression. Knockdown of SLC6A14 in pancreatic carcinoma significantly suppresses the activity of the Wnt/β-catenin pathway. It is hypothesized that SLC6A14 may further amplify Wnt signaling, establishing a malignant feedback loop of Wnt activation–nutrient acquisition–Wnt reactivation, thereby promoting tumor cell proliferation, metastasis, invasion, and EMT. The precise molecular mechanisms require further investigation [67].

The expression of SLC6A14 is upregulated in pancreatic and breast cancers, which activates mTORC1 signaling by increasing cellular arginine content [68,69]. Consequently, mTORC1 enhances the phosphorylation of 4EBP1 and thereby facilitates the translation of HIF-1α, which promotes tumor cell proliferation, migration, and invasion [52].

ATB0,+ and inducible nitric oxide synthase (iNOS) co-localize in cervical carcinoma specimens. So when ATB0,+ is significantly upregulated, arginine transport increases and NO production is sustained in cancer cells [70]. Additionally, NO can enhance MMP-1 transcription via the ERK and p38 MAPK pathways or promote MMP-2 transcription by increasing nuclear translocation of NF-κB and AP-1, resulting in extracellular matrix degradation and facilitating tumor invasion and metastasis [71,72]. SLC6A14 can transport NOS inhibitors, reducing NO synthesis by inhibiting iNOS activity [73].

In colorectal carcinoma, increased expression of ATB0,+ is observed predominantly on the apical and basolateral membranes of epithelial cells, accompanied by elevated iNOS levels. High levels of iNOS result in increased NO concentration within cancer cells [74]. Additionally, SLC6A14 activates the Janus kinase 2 (JAK2)/Signal Transducer and Activator of Transcription 3 (STAT3) signaling pathway, which enhances immunosuppression and colorectal carcinoma cell survival [75].

### 2.4. SLC38A4 (SNAT4)

SLC38A4, also named neutral amino acid transporter 4 (SNAT4), belongs to the SLC38 superfamily, which is a superfamily of transmembrane glycoproteins. SNAT4 features 10 transmembrane domains with both N- and C-termini oriented extracellularly, and the N-terminus undergoes glycosylation (Figure 6) [76]. It functions as a Na+-dependent neutral amino acid transporter and a Na+-independent cationic amino acid transporter, primarily transporting L-arginine and lysine. Due to the high affinity of cationic amino acids, it is likely the principal transporter responsible for cationic amino acid uptake in hepatic tissue [77]. The expression of SLC38A4 is predominantly observed in liver and placenta tissues [78,79]. However, studies indicate SNAT4 does not show significant accumulation of cationic amino acids, contradicting prior findings [80].

SLC38A4 functions as a tumor suppressor in hepatocellular carcinoma (HCC). SLC38A4 silencing promotes HCC cellular activity in vitro and tumorigenesis in vivo [78]. Downregulation of SLC38A4 enhances the β-catenin/c-Myc pathway, resulting in the suppression of 3-hydroxy-3-methylglutaryl-CoA synthase 2 (HMGCS2) expression [78,81]. HMGCS2 is a critical downstream target of SLC38A4, which could reverse the oncogenic roles of SLC38A4 depletion in HCC and suppress HCC cell proliferation and migration [82].

### 2.5. PEPT

The Peptide Transporter (POT) family comprises four members, each characterized by twelve transmembrane domains with both N- and C-termini oriented intracellularly. The POT family primarily mediates cellular uptake of dipeptides and tripeptides, functioning as H+-coupled symporters within the SLC15 transporter superfamily. The PEPT subgroup, which includes PEPT1 and PEPT2, encoded by the SLC15A1 and SLC15A2 genes respectively [83], shares approximately 70% sequence homology [84] and is predominantly localized to the plasma membrane. The POT family contains the PHT subgroup, which we will introduce later.

Peptide translocation can be defined as the process by which low-molecular-weight peptides, consisting of two to six residues, traverse cellular membranes [85]. The transmembrane transport of short peptides occurs across all biological systems as an efficient and energy-conserving pathway for amino acid uptake in peptide form. PEPT1 is an intestinal peptide transporter characterized by low affinity and high transport capacity; PEPT2 is a renal peptide transporter with high affinity and low transport capacity [83]. The PEPT transporter family exhibits broad substrate specificity, primarily mediating the transport of all possible dipeptides, tripeptides, and analogs, including dipeptides and tripeptides containing arginine, but not free amino acids [86]. These transporters are key targets in drug delivery, facilitating the uptake and tissue distribution of various important pharmaceutical compounds. Additionally, the high expression of PEPT1 in intestinal epithelial cells is extensively exploited in oral drug absorption [87,88]. The impact of free arginine internalized by small peptides on specific signaling pathways, its role in tumor progression, and whether it shares similarities with the above-mentioned transporters warrant further investigation.

It should be noted that the PEPT family also exhibits intracellular localization. Transport activity similar to PEPT1 has been observed within lysosomes of hepatic and renal tissues, as well as in the nucleus of vascular smooth muscle cells [89,90]. Its potential function may involve facilitating the efflux of oligopeptides from lysosomes into the cytosol, thereby maintaining lysosomal integrity, and failure to do so could result in pathological consequences [91]. Additionally, PEPT proteins have been identified on the mitochondrial membranes of PC3 (prostate cancer cell line) and U118 (glioblastoma cell line) cells [92].

Tumor-derived lactate induces the production of dipeptides and tripeptides via upregulation of MMPs and dipeptidyl peptidase IV (DPPIV) and stimulates PEPT1 by generating a H^+^ gradient across the plasma membrane [93]. The dipeptides transported by PEPT1 activate MAP4K4, a serine/threonine kinase, which phosphorylates Ras GTPase-activating protein binding protein 2 (G3BP2), promoting HCC proliferation, metastasis, and EMT. Higher histopathological grades of HCC samples correlate with increased PEPT1 expression, associated with reduced overall and disease-free survival rates [94].

SLC15A2 is upregulated in chronic myeloid leukemia. In chronic myeloid leukemia (CML) stem cells, SLC15A2 mediates dipeptide uptake and activates AMPK after internalization. This activation leads to phosphorylation of TSC2, subsequently activating Rheb or phosphorylating raptor to stimulate the mTORC1 pathway, thereby promoting tumor proliferation. Additionally, dipeptide internalization specifically activates the p38 MAPK pathway, resulting in phosphorylation of Smad3 at Ser208, which in turn activates Foxo3a, maintaining CML stem cell stemness and facilitating immune evasion. The mechanisms by which CML stem cells perceive internalized dipeptides and how p38 MAPK/Smad3/Foxo3a interact remain to be elucidated [95].

## 3. Intracellular Membrane Arginine Transporters

Current research on intracellular arginine transporters primarily focuses on mitochondrial and lysosomal membranes (Figure 2 and Table 2). Studies on other organelle membranes are relatively limited. For instance, SLC36A4 (PAT4) has been identified at the Golgi membrane, which is a transporter of small neutral amino acids [96]. PAT4 is also present at the plasma membrane [97] and lysosomal membrane [98]. Additionally, PHT2 has been discovered at the peroxisomal membrane, which facilitates the transport of free histidine and certain dipeptides and tripeptides [99]. PHT2 is also localized at lysosomal and endosomal membranes, which will be discussed in detail below. Future research may increasingly explore amino acid transporters at intracellular membranes.

### 3.1. Mitochondria

**Figure 7:**
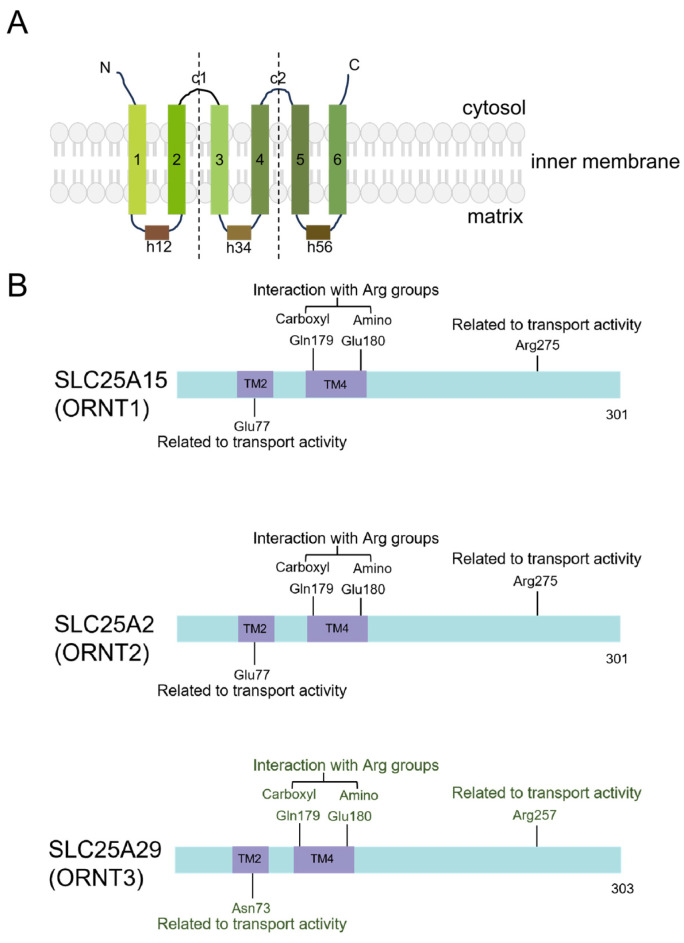
Topological and primary structure diagram of ORNT1/2/3. (**A**) SLC25 family can be recognized by their striking sequence features: a tripartite structure, six transmembrane α-helices, and three-fold repeated signature motifs. Each of three similar domains comprises two transmembrane alpha-helices, and the helices are linked by long matrix loops (h12, h34, and h56), and the domains are connected by shorter cytoplasmic loops (c1 and c2). (**B**) All listed loci are conserved within the ORNT enzyme family, showing comparable or analogous functions. Gln179 in ORNT1/2/3 is a substrate-specific site. Remarkably, regarding the residues on SLC25A29 associated with arginine translocation, current data are solely predictive and require empirical validation (green). TM, transmembrane region.

The human mitochondrial amino acid transporters belong to the SLC25 family, including SLC25A2 (ORC2, ORNT2), SLC25A15 (ORC1, ORNT1), and SLC25A29 (ORNT3), located at the mitochondrial inner membrane (Figure 2) [100]. The SLC25 family proteins are nuclear-encoded proteins, which consist of six transmembrane domains and three tandem repeats of conserved motifs with the N- and C-termini oriented toward the cytosolic side (Figure 7) [101,102].

#### 3.1.1. Ornithine Carriers (ORCs)

The ORCs include ORC1 and ORC2, respectively encoded by SLC25A15 and SLC25A2. ORC1 is expressed in the liver, pancreas, testes, lungs, and small intestine tissues and is responsible for the transport of L-arginine, L-ornithine, L-lysine, and L-citrulline. ORC2 is expressed in the liver, testes, spleen, lungs, pancreas, small intestine, and kidneys tissues and is responsible for the transport of L/D-arginine, L/D-ornithine, L/D-lysine, L/D-histidine, asymmetric dimethyl L-arginine (ADMA), as well as L-citrulline and L-homoarginine. ORC is a H^+^-driven antiporter and mainly transports arginine to cytoplasm. It serves as a critical metabolic hub, regulating excessive arginine catabolism [103,104,105].

SLC25A15 may play a tumor suppressor role in HCC. Glutamine and arginine metabolism overlap in mitochondria. Once imported into mitochondria, glutamine releases NH_4_^+^ [106]. So, when SLC25A15 deficiency leads to glutamine reprogramming, it results in ammonia accumulation, indicating urea cycle dysfunction. We speculate that it may disrupt arginine metabolism homeostasis. And patients with low expression of SLC25A15 have a poor prognosis [107,108].

#### 3.1.2. SLC25A29 (ORNT3)

SLC25A29, also known as ORNT3, is a mitochondrial cationic amino acid transporter primarily responsible for the translocation of arginine and lysine to mitochondria. This progress is reverse transport and unidirectional transport. The protein is expressed across various tissues, with higher expression levels observed in the brain and heart [109,110].

SLC25A29 is a prognostic biomarker associated with poor outcomes in prostate cancer. Upregulation of SLC25A29 may activate the β-catenin/c-Myc/E2F1 signaling pathway, leading to E2F1 binding to the promoter region of DNA polymerase delta 1 (POLD1), thereby reducing oxidative stress in cancer cells and maintaining mitochondrial function. Meanwhile, SLC25A29 may facilitate arginine transport and maintain mitochondrial NO homeostasis, which thereby supports mTOR signaling pathway transduction [111,112]. SLC25A29 may also inhibit the recruitment and activation of anti-immune cells, which contributes to tumor immune evasion [113]. In lung adenocarcinoma, the downregulation of SLC25A29 is associated with lactate produced by cancer cells. Lactate can induce lactylation modifications in the promoter region of SLC25A29, which suppresses the transcription of SLC25A29 and thereby promotes endothelial cell survival and migration [110].

### 3.2. Lysosome

#### 3.2.1. SLC38A9 (SNAT9)

SNAT9, encoded by SLC38A9, functions as a lysosomal membrane transporter, which is characterized by 11 transmembrane domains with the N-terminus located in the cytosolic lumen and the highly glycosylated C-terminus within the lysosomal lumen (Figure 2) [114]. It is ubiquitously expressed across tissues [115] and mediates the efflux of essential amino acids (including leucine, phenylalanine, isoleucine, tryptophan, and methionine) and arginine from the lysosome into the cytoplasm. However, its affinity for arginine transport is low, which cannot obviously regulate lysosomal arginine levels [116]. Arginine is transported via SNAT9, which is coupled to the activation of mTORC1 and lysosomal amino acid trafficking. Upon arginine stimulation, SNAT9 interacts directly with mTORC1 through Rag GTPases, localizing to the lysosomal surface to initiate autophagy signaling [117,118].

SLC38A9 could also serve as a lysosomal arginine sensor for mTORC1 regulation. Arginine depletion may activate nuclear factor erythroid 2-related factor 3 (NRF3), and thereby induce SLC38A9 expression, which promotes mTORC1 recruitment onto lysosomes. NRF3-mediated arginine-dependent mTORC1 activation results in tumor growth and poor prognosis [119].

#### 3.2.2. SLC66A1 (PQLC2)

PQLC2, encoded by SLC66A1, is a lysosomal membrane protein characterized by seven transmembrane domains. Its glycosylated N-terminus is oriented to the lysosomal lumen (Figure 2). The C-terminus is localized in the cytoplasm, which determines its lysosomal localization. Unlike other lysosomal transporters, PQLC2 is decoupled from the H+ gradient, but its transport activity depends on the acidic lysosomal lumen. PQLC2 has a broad substrate specificity and can transport cationic amino acid unidirectionally from the lysosomal lumen to the cytoplasm, and it is a principal arginine transporter [120,121,122].

PQLC2 could also function as a transporter receptor, which is different from the amino acid sensing mechanism of SNAT9. Instead, it is able to recruit the C9orf72-SMCR8-WDR41 (CSW) complex to the lysosome membrane when the levels of cationic amino acid in lysosome are decreased, which may thereby enhance mTORC1 activity [123].

PQLC2 was highly expressed in gastric carcinoma tissues, which is positively associated with poor patient survival. Overexpression of PQLC2 enhances cell proliferation and tumor formation in nude mice by activating MEK/ERK1/2 and PI3K/AKT signaling, suggesting that PQLC2 is a potential target [124]. Whether PQLC2 functions as an amino acid transporter in this progression needs further investigation.

#### 3.2.3. SLC7A14

SLC7A14 is a lysosomal membrane protein and an orphan protein of the SLC7 transporter family. It is predominantly expressed in dermal fibroblasts, neurons, and primary endothelial cells [125,126]. The protein facilitates the import of gamma-aminobutyric acid (GABA) into lysosomes, thereby promoting insulin resistance [127]. Additionally, it may mediate the translocation of cationic amino acids from the lysosomal membrane to cytoplasm, which may contain arginine [125].

#### 3.2.4. PHT1/2

The PHT family belongs to the peptide transporter (POT) superfamily, comprising PHT1 and PHT2, respectively encoded by SLC15A4 and SLC15A3, which are localized on lysosomal and endosomal membranes.

The PHT family is expressed across various tissues and organs, including the intestine, eyes, spleen, lungs, and thymus. PHT1 is predominantly expressed in the nervous and immune systems, facilitating the lysosomal transport of free histidine and certain dipeptides and tripeptides into the cytosol [128,129]. Similar to PEPT, the role and mechanism of isolated arginine remain unresolved. But one point is certain: members of the SLC15 family have been identified as relevant pharmacological targets at the level of drug transport in different cellular locations [84].

Mitochondria and lysosomes are central to arginine metabolism and signal transduction [106,130]. We found that the role of membrane transport proteins in mediating arginine delivery into organelles remains poorly understood, whereas most existing studies have focused primarily on alterations in metabolic enzymes. For example, normal cells synthesize ornithine via OAT, whereas cancer cells rely exclusively on mitochondrial arginase 2—an enzyme highly expressed in specific tumor types [131]. Cancer cells can augment protumor metabolic flux through two distinct mechanisms: the upregulation of specific arginine transporters localized to mitochondria or lysosomes, and the modulation of their subcellular distribution. Notably, although SLC7A5 does not function as an arginine transporter, it may serve as the exclusive pathway to supply adequate citrulline for de novo arginine synthesis in tumors [132]. Meanwhile, researchers have observed that macrophages are capable of taking up arginine secreted by cancer cells [133]. Collectively, these observations reveal that cancer cells exert precise control over arginine distribution, a process likely mediated by an undefined organelle membrane transport machinery. Given that the molecular mechanisms linking mitochondria/lysosomes to arginine transport remain poorly characterized, this represents a critical knowledge gap that merits in-depth exploration.

## 4. Arginine Uptake-Targeted Strategies for Tumor Therapy

Arginine-dependent tumors, such as colorectal cancer, melanoma, and non-epithelioid pleural mesothelioma, are deficient in urea cycle enzymes (OTC, ASS, ASL), rendering them highly reliant on extracellular arginine uptake [134,135,136,137,138,139]. Depletion of arginine to concentrations below the functional threshold induces compensatory amino acid transport and adaptive cellular responses via arginine deprivation [140,141,142]. These cells exhibit a marked dependence on extracellular arginine for survival and are typically sensitive to arginine deprivation [142]. Therefore, arginine deprivation therapy is viable, with its efficacy contingent upon the status of endogenous synthesis pathways and cellular uptake capacity [143].

The extracellular arginine supply to tumor cells can be abrogated through dietary restriction, arginine-degrading enzymes, and arginine transport inhibition. Arginine-degrading enzymes include NOS, ARG, AGAT, ADC, and mycoplasma-derived arginine deiminases (ADIs). Recombinant enzymes such as PEGylated ADI (ADI-PEG20) and PEGylated mutated human arginase I (PEG-BCT-100) exhibit low immunogenicity, toxicity, and extended half-life, demonstrating clinical efficacy against arginine-dependent tumors [141,144]. In preclinical studies, ADI-PEG20 effectively inhibits the progression of non-epithelial mesothelioma, melanoma, and hepatocellular carcinoma and is now being clinically investigated as an arginine deprivation therapy for these tumors [134,136]. PEG-BCT-100 exhibits favorable efficacy and safety profiles in combined treatment of hepatocellular carcinoma, pancreatic cancer, melanoma, and prostate cancer [139,145,146].

An alternative approach to arginine deprivation involves disrupting arginine uptake. As the growth of certain arginine-dependent tumor cells relies on the upregulation of specific arginine transporters, these transporters exhibit markedly higher expression levels in tumors relative to normal cells, thereby highlighting their potential as promising targets for cancer therapy (Table 3). Most drugs targeting the SLC family relate to arginine transport and energy metabolism [147,148]. For example, arginine analogs or derivatives, including L-lysine, L-ornithine, L-N^ω^-methylargine, and asymmetric dimethylarginine (ADMA), can be used as arginine inhibitors to compete for transporter binding sites [148,149]. α-Methyltryptophan (α-MT) can act as an inhibitor of SLC6A14, induce arginine deprivation, suppress mTOR signaling, and selectively promote autophagy in colorectal cancer cells [65]. Cell surface transporter proteins can be inhibited via monoclonal antibodies or non-coding RNAs. For instance, anti-CAT-1 monoclonal antibody suppresses human colorectal cancer tumor growth [19]. SLC7A1 acts as a downstream target of miR-122; notably, PD407824 (a Wee1 kinase inhibitor) and Ellipticine (a DNA topoisomerase inhibitor) can enhance miR-122 expression levels, which in turn decreases SLC7A1 expression [150,151,152].

Arginine depletion impairs tumor cell survival and exerts adverse effects on immune cells within the tumor microenvironment [148]. Thus, enhancing the specificity of chemotherapeutic agents toward malignant cells is necessary. As drug absorption is modulated by transporter proteins, most of these proteins represent promising druggable targets for cancer therapy [126,153]. Transporter proteins mediate the cellular uptake of drugs to facilitate their therapeutic effects and can also actively efflux drugs out of cells, thereby contributing to chemoresistance. For example, CAT-2 facilitates intracellular delivery of anticancer drugs [27]. Among numerous arginine transporters, ATB0,+ and the PEPT subgroup display broad substrate specificity, rendering them ideal targets for chemotherapeutic drug delivery in tumor cells. Anticancer agents can be rationally designed or chemically modified to serve as substrates for these transporters, thereby enabling targeted delivery to tumor cells [154]. Leveraging amino acids as linker moieties in prodrug design enables the development of optimized formulations of existing pharmaceuticals, which are referred to as amino acid prodrugs [155,156]. First, prodrugs can enhance the aqueous solubility and transporter-protein-mediated permeability of drugs, thereby increasing PEPT1-mediated intestinal absorption of oral polar pharmaceuticals, such as gemcitabine and quercetin [157,158]. Second, prodrugs can improve the targeting efficacy of the parent drug. By increasing the affinity for transporter proteins and encapsulating prodrugs within nanoparticle carriers, their activation can be achieved within target tissues and organs in response to various stimuli, including pH, reactive oxygen species (ROS), glutathione (GSH), light, and heat [156,159]. For example, co-encapsulating CD98 siRNA (siCD98) and camptothecin into CD98 Fab-functionalized nanoparticles enables the simultaneous and efficient delivery of both agents to CD98-overexpressing target cancer cells and tumor tissues [159,160].

Cancer cells characteristically express multiple arginine transporters with overlapping functional profiles, which poses a major obstacle to the development of highly selective inhibitors targeting specific SLC family transporters. In addition, arginine transporter expression patterns display remarkable inter- and intracancer type heterogeneity. Even with the notable progress summarized above, especially regarding the optimization of drug delivery specificity, safety, and stability, the road ahead for translational research in this area remains long and arduous.

## 5. Conclusions

In this review, we systematically summarize the structural characteristics, functional roles, and involvement pathways of these transporters under pathological conditions, primarily in tumorigenesis. Our findings reveal that the majority of arginine transporter complexes facilitate arginine translocation. In other words, arginine activates mTORC1 signaling and its downstream pathways or stimulates Wnt/β-catenin signaling and positive feedback loops, thereby amplifying Wnt signaling and exerting oncogenic functions. Consequently, compared to the broader metabolic reprogramming of arginine-related pathways in cancer, the transporters themselves may hold greater significance. Their cellular localization and structural features determine their functional roles, profoundly influencing the fate of cancer cells.

## Figures and Tables

**Table 1:** Cell membrane arginine transporters.

Gene Name	Protein Name	Substrate	Expression	Ion Dependence
*SLC6A14*	ATB0,+	All essential amino acids	Expressed in the apical membranes of respiratory tract, salivary glands, mammary glands, stomach, pituitary, ocular tissues, and distal intestinal tissues	Coupled Na+ and Cl− gradient
*SLC7A1*	CAT-1	Cationic amino acids (ornithine, arginine, and lysine)	Expressed extensively throughout the human body except in the liver	Non-Na+ coupling
*SLC7A2*	CAT-2	Cationic amino acids	CAT-2A is predominantly constitutively expressed in hepatic tissue, whereas CAT-2B is typically inducible across various cell types under inflammatory conditions	Non-Na+ coupling
*SLC7A3*	CAT-3	Cationic amino acids	Expressed in various regions of the human brain and numerous peripheral tissues	Non-Na+ coupling
*SLC7A6* *+ SLC3A2*	yLAT2+4F2hc	Cationic amino acids	Expressed in brain and lung tissues	Non-Na+ coupling
*SLC7A7* *+ SLC3A2*	yLAT1+4F2hc	Cationic amino acids	Expressed in brain, heart, testis, kidney, and small intestine tissues	Non-Na+ coupling
*SLC7A9* *+ SLC3A1*	b0,+AT+rBAT	Cationic amino acids	Expressed in small intestinal epithelial cells	Non-Na+ coupling
*SLC38A4*	SNAT4/ATA3	Mainly L-arginine and lysine	Mainly expressed in liver and placenta tissues	Non-Na+ coupling

**Table 2:** Intracellular membrane arginine transporters.

Gene Name	Protein Name	Substrate	Subcellular Localization	Ion Dependence
*SLC7A14*		Cationic amino acid	Lysosome and endosome	H+ propulsion
*SLC25A2*	ORC2/ORNT2	L/D-ornithine, lysine, arginine, histidine, L-citrulline, and L-homoarginine	Mitochondrion	H+ propulsion
*SLC25A15*	ORC1/ORNT1	L-ornithine, L-lysine, L-arginine, and L-citrulline	Mitochondrion	H+ propulsion
*SLC25A29*	ORNT3	Mainly arginine and lysine	Mitochondrion	H+ propulsion
*SLC38A9*	SNAT9	Low affinity for arginine transport	Lysosome	Independent
*SLC66A1*	PQLC2	Arginine, lysine, histidine, and ornithine	Lysosome	Independent

**Table 3:** Arginine transporters in cancers.

Transporter	Subcellular Localization	Cancer	Expression	Regulatory Pathway	Effect	Clinical Significance	References
SLC3A1	Cell membrane	Breast cancer	Upregulated in tumor tissues	Elevated arginine uptake activates the AKT/GSK3β/β-catenin axis	Promote tumorigenesis	Overexpression is correlated with advanced clinical stages and poor patient survival	[60]
SLC6A14	Cell membrane	Cervical cancer	Upregulated in tumor tissues	Elevated arginine uptake supports the activity of iNOS	/	/	[70]
SLC7A1	Cell membrane	Colorectal cancer	Upregulated in cancer-derived extracellular vesicles (EVs)	Elevated arginine uptake activates downstream NO metabolic cascade in vascular endothelial cells	Promote tumor angiogenesis	Plasma EV-SLC7A1 is elevated in colorectal cancer patients	[21]
SLC7A1	Cell membrane	Ovarian cancer	Upregulated in cancer cells and cancer-associated fibroblasts (CAFs)	SLC7A1 is involved in activating MAPK/ERK signaling and EMT	Promote cancer metastasis and resistance to cisplatin	Overexpression is correlated with poor patient survival	[20,22]
SLC7A2	Cell membrane	Pancreatic ductal adenocarcinoma	/	RIOK3 promotes mTORC1 activation by facilitating SLC7A2-mediated arginine uptake	Involved in RIOK3-induced cancer progression	/	[24]
SLC7A2	Cell membrane	Hepatocellular carcinoma	Downregulated in tumor tissues	Deficient SLC7A2 mediated the upregulation of CXCL1 through PI3K/Akt/NF-κB pathway to recruit myeloid-derived suppressor cells (MDSCs)	Exert tumor immunosuppressive effect	Negative expression is correlated with increased tumor size, advanced stage, and poor patient survival	[29]
SLC7A3	Cell membrane	Osteosarcoma	Upregulated in tumor tissues	SIRPA-SP1-SLC7A3 axis enhances SP1 stabilization cycle via promoting arginine uptake	Promote osteosarcoma metastasis	Positively correlated with epithelial–mesenchymal transition (EMT) marker	[33]
SLC7A7	Cell membrane	Hepatocellular carcinoma	/	ATF3-SLC7A7 axis suppresses mTORC1 signaling	Suppress lipogenesis and tumorigenesis	/	[46]
SLC7A7	Cell membrane	T-cell acute lymphoblastic leukemia	Upregulated in the bone marrow samples of children with T-ALL	Arginine efflux induced by SLC7A7 inhibits mTOR protein expression	Promote cell viability	/	[47]
SLC25A29	Mitochondrial inner membrane	Prostatic cancer	Upregulated in tumor tissues	SLC25A29 transactivates POLD1 via E2F1 by transporting arginine into mitochondria	Promote tumor progression	Overexpression is correlated with metastatic features and poor patient survival	[111]
SLC38A9	Lysosomal membrane	Multiple cancers	Upregulated in tumor tissues	NRF3 induces arginine-dependent mTORC1 recruitment onto lysosome via SLC38A9	Promote tumor progression	Overexpression is correlated with poor patient survival	[119]

## Data Availability

No new data were created or analyzed in this study. Data sharing is not applicable to this article.
